# Impact of Bonding Pressure on the Reactive Bonding of LTCC Substrates

**DOI:** 10.3390/mi16030321

**Published:** 2025-03-11

**Authors:** Erik Wiss, Nesrine Jaziri, Jens Müller, Steffen Wiese

**Affiliations:** 1 Chair of Microintegration and Reliability, Faculty of Natural Sciences and Technology, Saarland University, 66123 Saarbrücken, Germany; erik.wiss@uni-saarland.de; 2 Electronics Technology Group, Faculty of Electrical Engineering and Information Technology, TU Ilmenau, 98693 Ilmenau, Germany; nesrine.jaziri@tu-ilmenau.de (N.J.); jens.mueller@tu-ilmenau.de (J.M.)

**Keywords:** LTCC, reactive multilayers, RMS, Al/Ni, bonding

## Abstract

Reactive bonding can overcome the issues associated with conventional soldering processes, such as potential damage to heat-sensitive components and the creation of thermomechanical stress due to differing coefficients of thermal expansion. The risk of such damage can be reduced by using localized heat sources like reactive multilayer systems (RMS), which is already a well-established option in the field of silicon or metal bonding. Adapting this process to other materials, such as low temperature co-fired ceramics (LTCC), is difficult due to their differing properties, but it would open new technological possibilities. One aspect that significantly affects the quality of the bonding joints is the pressure applied during the bonding process. To investigate its influence more closely, various LTCC samples were manufactured, and cross-sections were prepared. The microscopical analysis reveals that there is an optimum range for the bonding pressure. While too little pressure results in the formation of lots of voids and gaps, most likely in poor mechanical and electrical properties, too high pressure seems to cause a detachment of the metallization from the base material.

## 1. Introduction

Reflow soldering is the standard process in the manufacturing of electronic assemblies, particularly for surface-mount technology (SMT) applications. This process begins with the application of solder paste, composed of powdered solder and flux, to the contact pads of a printed circuit board (PCB). In the next step, components are placed onto these pads on the PCB using automated pick-and-place machines, ensuring accurate positioning. The assembled PCB is then passed through a reflow oven, where it is subjected to a specified heating profile, typically involving two temperature zones: a preheating zone below the melting point of the solder paste, and a reflow zone above the melting point of the solder paste. The preheating zone ensures uniform heating of the PCB and all components, while the reflow zone melts the solder paste, creating a proper mechanical and electrical connection by forming an intermetallic compound (IMC). Although reflow soldering is a relatively simple and quick process, it has some disadvantages primarily attributed to global heat exposure. These include the risk of damage to heat-sensitive components and the development of thermomechanical stress due to differing coefficients of thermal expansion (CTE) between the components and the base material, potentially leading to failures. A new approach to preventing or minimizing such damage involves using localized heat sources instead of global ones, such as the exothermic reaction of reactive multilayer systems (RMS) [1,2,3].

**Figure 1:**
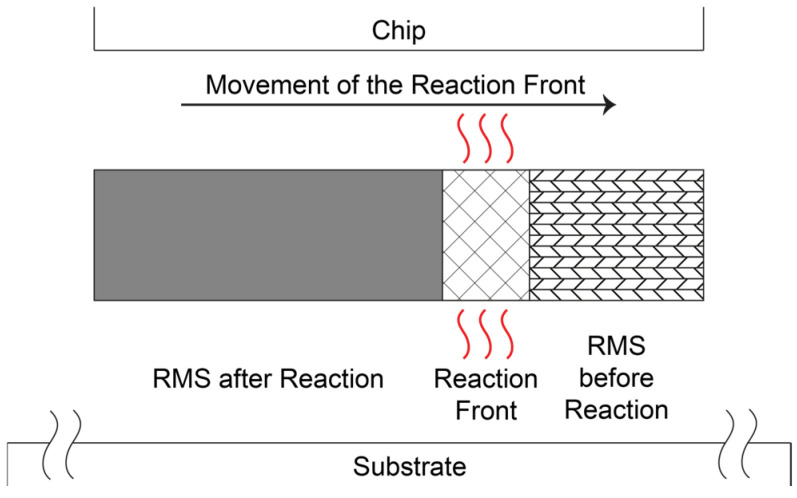
The fundamental principle of the usage of a reactive multilayer system (RMS) for bonding processes in electronics packaging. In the initial state, the materials are arranged in alternating nanolayers. Ignition, for example, by a laser pulse or an electric spark, causes intermixing of the materials. The heat released from this exothermic reaction can be used for a localized soldering process.

An RMS consists of numerous nanoscale bilayer stacks of at least two different reactant partners, with a total stack thickness of up to 300 µm [4,5]. Some possible material combinations are Ni and Al, Ti and Al, Nb and Si, or B and Ti [6,7,8,9]. When supplied with sufficient energy, for example through a laser pulse or an electric spark, the materials react with each other to form an IMC. Since the enthalpy of this reaction is negative, it is exothermic [10], meaning that energy is released in the form of heat, which can be used to locally melt the solder material. The released heat is so substantial that the reaction is self-sustaining and continues until all the available material has been converted. Further, the reaction occurs within fractions of a second; depending on the material system and layer thicknesses, velocities of more than 10 m/s can be achieved. Other potential future applications of RMS could include self-repairing solder joints or the large-scale removal of components for recycling purposes. The basic principle of the heat release of an RMS is shown in Figure 1.

The application of RMS is already a well-established process in the field of silicon or metal bonding [11,12,13]. However, adapting this process to other base materials, such as conventional PCB material or low-temperature co-fired ceramics (LTCC), is technologically challenging due to their much higher roughness and significantly lower thermal conductivity [14,15]. Nevertheless, this adaptation would open new possibilities, particularly in the area of heterogeneous integration. Therefore, a better understanding of the reaction in combination with these materials is necessary. Previous studies have already addressed the deposition process of RMS on various pre-treated LTCC surfaces [16,17,18], the influence of additional solder layers on the evolving temperatures [19], and the influence of bonding pressure on both the reactive joining of metals [20] and the thermal resistance of Si chips [21]. In this study, the influence of bonding pressure on the quality of the bonding joints will be investigated and compared.

## 2. Materials and Methods

### 2.1. Sample Preparation

In order to investigate the influence of the bonding pressure on the quality of the bonding zones, different bonding pressures were used to manufacture the samples. All samples were manufactured of a widely used LTCC material, GreenTape 951 (DuPont, Wilmington, DE, USA), in combination with a suitable Pd/Ag thick-film paste 6146 (DuPont) for metallization. A size of 3 mm × 3 mm, matching the tool size of the bonding machine (see Section 2.2), was chosen for the chips. The Pd/Ag metallization was applied to the entire surface of Samples L1–L5, and only to four small pads measuring 0.5 mm × 0.5 mm in the corners of samples L6–L10 (see Table 1). This reduction in the effective bonding area allowed much higher bonding pressures to be achieved. The substrates had a size of 15 mm × 3 mm to allow easier embedding by matching the size of the embedding forms. Here, the Pd/Ag metallization was applied centrally, either as a single area or as individual pads, depending on the respective variant. After sintering under standard LTCC processing conditions, thicknesses of approximately 840 µm for the substrate and 10 µm for the metallization were achieved.

**Figure 2:**
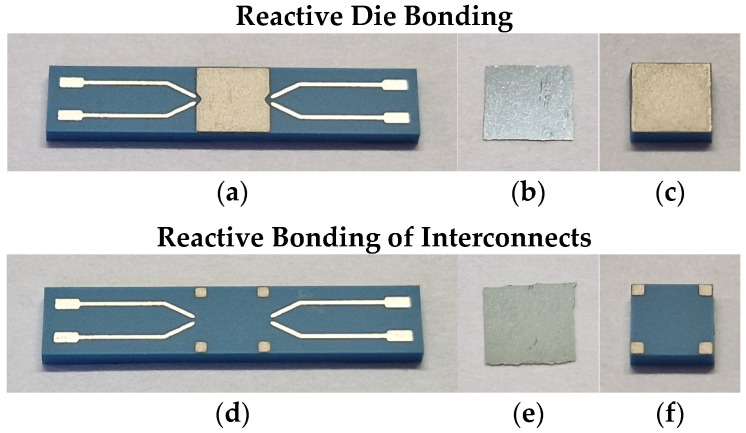
(**a**–**c**) Reactive die bonding. (**a**) LTCC substrate (15 mm × 3 mm) with full-area Pd/Ag metallization. (**b**) Reactive foil with Sn coating on both sides. (**c**) LTCC chip (3 mm × 3 mm) with full-area Pd/Ag metallization. (**d**–**f**) Reactive bonding of interconnects. (**d**) LTCC substrate (15 mm × 3 mm) with Pd/Ag metallization on four corner pads (0.5 mm × 0.5 mm). (**e**) Reactive foil with Sn coating on both sides. (**f**) LTCC chip (3 mm × 3 mm) with Pd/Ag metallization on four corner pads (0.5 mm × 0.5 mm).

A commercially available reactive foil NanoFoil^®^ NF40 (Indium Corp., Clinton, NY, USA) was used for the bonding process. As described in the introduction, it consists of numerous bilayers of aluminum and nickel with a total stack thickness of approximately 40 µm. The individual layers also contain small amounts of vanadium, indium, silver, and copper to enhance the reaction properties, such as mechanical stability and electrical and thermal conductivity. Both sides of the foil are pre-coated with an approximately 5 µm thick layer of tin, eliminating the need for additional solder paste. The foil was cut into small pieces with a sharp scalpel. Their size was chosen to be slightly larger than the Pd/Ag areas to simplify the manual placement process and to be able to reach them easily with the ignition module. Figure 2 shows the LTCC samples and the reactive foils.

### 2.2. Bonding Process

**Figure 3:**
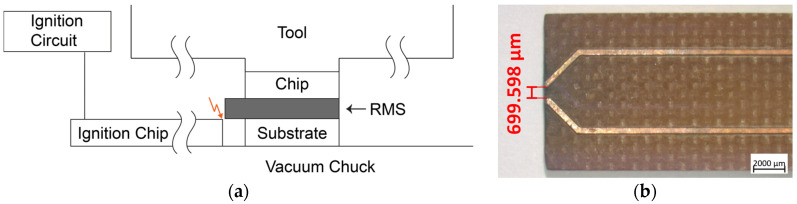
(**a**) Schematic side view of the bonding setup. The tool head uses a vacuum mechanism to pick up the chip. After aligning with the substrate, the RMS foil is positioned on the substrate with a slight protrusion towards the ignition chip. Then, the bonding process is initiated, during which the tool presses the chip onto the substrate with a defined force. After reaching the set parameters, the ignition chip is manually brought close to the foil, and the ignition is triggered. (**b**) Ignition chip with two converging tracks at the edge, separated by a gap of approximately 700 µm. The electric spark generated between the tracks upon triggering the ignition circuit can be used to ignite the RMS.

The bonding process was carried out on a die bonder FINEPLACER pico (Finetech, Berlin, Germany), which has already proven its suitability for bonding processes using RMS several times [22,23]. The main components are a vacuum chuck with an integrated heater, a tool with an integrated vacuum holder and heater mounted on a movable arm, a force module to exert a specific force of up to 400 N on the bonding partners, and a visual alignment system consisting of two cameras. Figure 3a shows the schematic side view of the bonding setup.

The ignition circuit transforms a low voltage of 9 V from a battery to approximately 15 kV, and is connected to an ignition chip with two tracks that converge at its edge with a small gap of approximately 700 µm between them (see Figure 3b). As the quotient of the voltage and the gap is greater than the breakdown field strength of air (3 kV/mm for dry air and standard pressure), an electrical spark is generated when the ignition circuit is triggered. This spark has a sufficient amount of energy to initiate the start of the reaction of the RMS.

**Figure 4:**
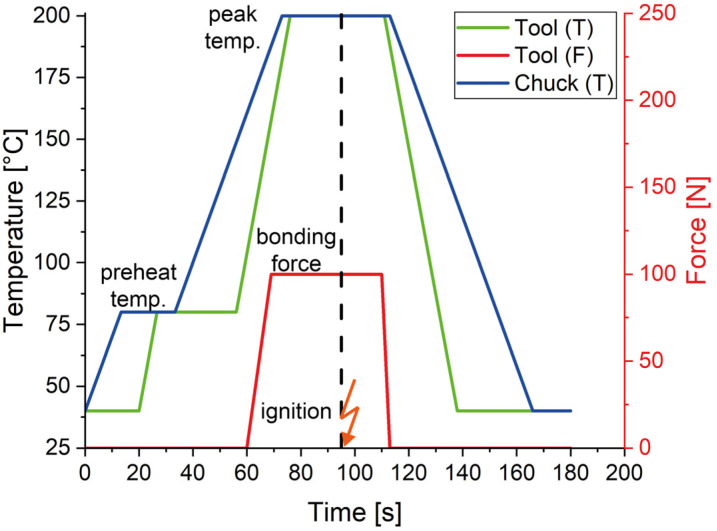
Idealized time–temperature (green: tool, blue: vacuum chuck) and time–force (red) profiles of the die bonder for the LTCC samples. While the force was set to 100 N, the preheat and maximum temperature were set to 80 °C and 200 °C, respectively. The vertical dashed line marks the timestamp of the ignition of the reactive foil.

The substrate was placed on the vacuum chuck, and the chip was picked up by the tool using a vacuum. The videos from both cameras, one pointing at the substrate and one at the chip, were overlapped to allow very precise alignment of the bonding partners. The cut RMS foil was then placed on the substrate with a slight protrusion towards the ignition chip, and the actual bonding process was initiated. For the temperature profile, a preheat temperature of 80 °C and a peak temperature of 200 °C were chosen. The target temperature on the tool corresponds to that of the chuck, but it requires more time to heat up due to its much greater thermal mass. After the preheating phase was completed, the arm was automatically moved downwards, placing the chip on the substrate, and the force module started to apply the force, and thus a pressure, on the bonding partners. Table 2 summarizes the different sample configurations regarding the bonding profiles and the resulting bonding pressures, and Figure 4 exemplarily shows the course of the bonding profile with a bonding force of 100 N.

**Figure 5:**
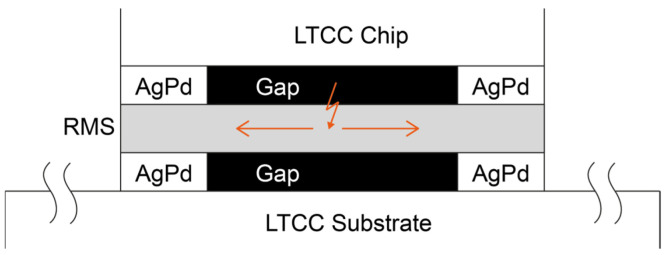
The ignition of the foil is initiated approximately in its center, causing the reaction to propagate outward.

Once the set force was reached, the ignition chip was manually moved to the side of the foil and the ignition process was initiated. The foil is ignited approximately in the center, causing the reaction to propagate outward (see Figure 5).

### 2.3. Microscopical Analysis

Cross-sections of the samples were prepared in several steps to facilitate light microscopy, scanning electron microscopy (SEM), and energy dispersive X-ray spectroscopy (EDX) analyses. These techniques were used to evaluate the quality of the bonding zones and to estimate their mechanical and electrical properties. Initially, the samples were embedded in epoxy resin using an EpoFix kit (Struers, Ballerup, Denmark), and cured for 24 h under slight negative pressure to remove air bubbles. Next, plane and fine grinding were performed on a LabPol-25 (Struers) using SiC foils with grits of 500 and 1200. Diamond polishing followed on a Tegramin-25 (Struers) using suspensions with particle sizes of 9 µm, 3 µm, and 1 µm. The samples were then subjected to final polishing with a 0.25 µm silicon oxide suspension on a Saphir Vibro (ATM Qness, Mammelzen, Germany) vibratory polishing machine to obtain an extremely flat surface with ultra-low roughness. Lastly, a thin carbon layer of approximately 20 nm thickness was applied using a Q150T Coater (Quantum Design, Pfungstadt, Germany) to prevent charging effects during SEM analysis caused by electron accumulation or the formation of space–charge regions. Light microscopy analysis using an Axio Imager.M2m (Zeiss, Oberkochen, Germany), equipped with a 20× objective was performed to obtain stitched images of the samples. The SEM analysis was carried out on an EVO MA15 (Zeiss) equipped with a LaB_6_ cathode, operating at an accelerating voltage of 20 kV and a beam current of 40 µA. EDX analysis utilized an XFlash detector (Bruker, Billerica, MA, USA) with an energy resolution of 123 eV.

## 3. Results

### 3.1. Light Microscopy

The image stitching method was used first to obtain an overall view of the bonding zones of every sample. Multiple individual images were combined into a single composite image, allowing the entire bonding zone to be displayed in high resolution. This approach simplifies the direct comparison of the samples with respect to properties such as gap or crack formation, solder contribution, and integrity of the metallization.

**Figure 6:**
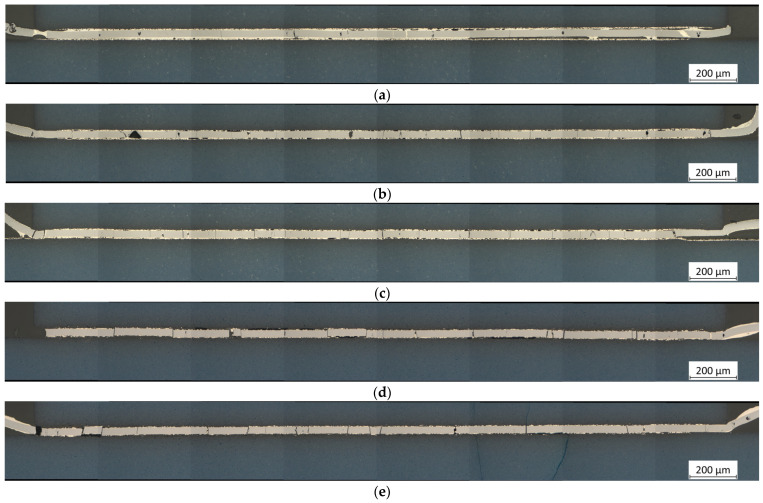
Stitched images of LTCC samples L1–L5 with full-area Pd/Ag metallization (blue: LTCC material; gray: RMS after the reaction; white-yellow: solder; red-yellow: metallization). The samples were bonded under pressures of approximately (**a**) 2 MPa, (**b**) 11 MPa, (**c**) 22 MPa, (**d**) 33 MPa, and (**e**) 44 MPa. Bonding at low pressures results in the formation of numerous gaps and poor adhesion between the Pd/Ag metallization and the solder. Medium pressures reduce the number of gaps and voids, thereby improving the overall performance. However, if the pressure is too high, the RMS experiences significant thermo-mechanical stress, leading to multiple fractures that remain predominantly unfilled. At the highest pressure, fractures are even generated within the LTCC material. Magnification: 20×.

**Figure 7:**
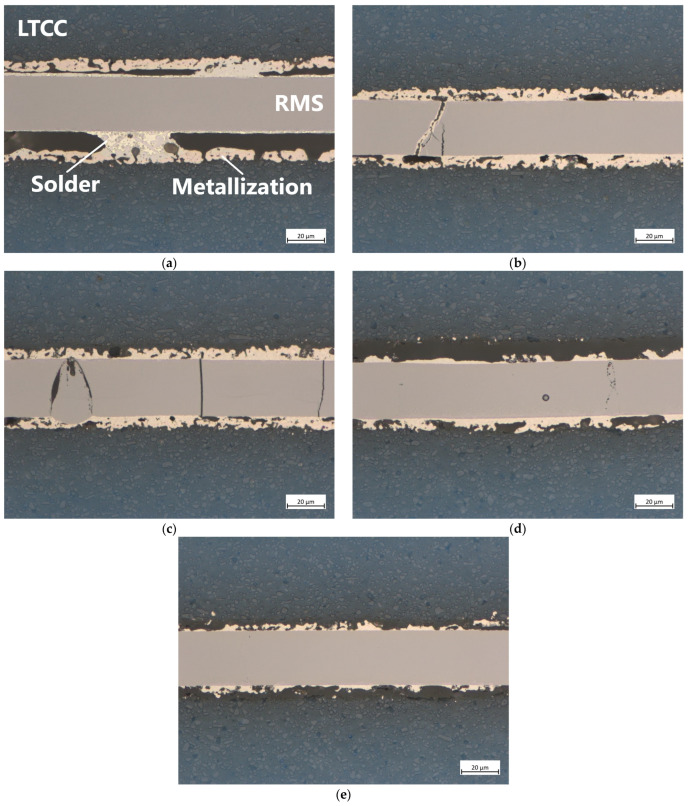
Detailed pictures of LTCC samples L1–L5 with full-area Pd/Ag metallization (blue: LTCC material; gray: RMS after reaction; white-yellow: solder; red-yellow: metallization). The samples were bonded under pressures of approximately (**a**) 2 MPa, (**b**) 11 MPa, (**c**) 22 MPa, (**d**) 33 MPa, and (**e**) 44 MPa. Magnification: 50×.

Figure 6 shows the stitched images of samples L1–L5 with full-area Pd/Ag metallization, bonded under pressures of approximately 2 MPa (L1), 11 MPa (L2), 22 MPa (L3), 33 MPa (L4), and 44 MPa (L5). Figure 7 presents more detailed images at 50× magnification, representative of the respective samples. Figure 6a shows that using a very low bonding pressure of 2 MPa results in a very uneven joint. While the left-hand side and the center appear relatively decent, the right-hand side exhibits significantly poorer connection, characterized by large gaps between the metallization and the RMS, as well as the solder. Additionally, some cracks have formed in the reactive layer, which, in a few cases, have been filled with solder. Figure 7a shows a location with poor wetting behavior of the solder, which appears to accumulate in a few spots, except for a very thin layer on the RMS. The metallization and the solder are clearly distinguishable as separate layers throughout the entire area.

Increasing the bonding pressure to 11 MPa results in a much better appearance than before (Figure 6b and Figure 7b). Although many gaps are still visible, they have become smaller and less extensive. The number of cracks in the RMS, most of which remain unfilled, has increased. The more detailed image reveals that the bonding zone is narrower than before, offering the possibility of improved formation of an IMC between the solder and the metallization. A further increase in bonding pressure to 22 MPa leads to similar results (Figure 6c and Figure 7c). Compared to the bonding pressure of 2 MPa, the gaps become smaller, and the number of cracks has increased. Overall, the bonding zone looks the best here.

Significant differences become visible when the pressure is increased to 33 MPa (Figure 6d and Figure 7d) or even 44 MPa (Figure 6e and Figure 7e). The high pressure, in combination with the high temperatures of the reaction, causes the RMS to break at several points, forming separate blocks. In the case of the highest pressure, cracks were also found in the LTCC material. As before, most of the cracks in the RMS remain unfilled. Judging by the more detailed images, the metallization layers were also damaged here.

**Figure 8:**
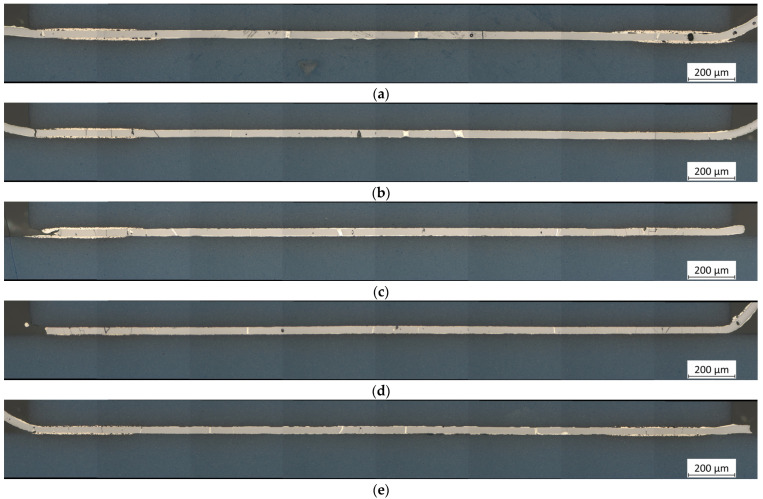
Stitched images of LTCC samples L6–L10 with Pd/Ag metallization on four corner pads of size 0.5 mm × 0.5 mm (blue: LTCC material; gray: RMS after reaction; white-yellow: solder; red-yellow: metallization). Compared to the previous samples, the applied pressures of (**a**) 20 MPa, (**b**) 100 MPa, (**c**) 200 MPa, (**d**) 300 MPa, and (**e**) 400 MPa are significantly higher due to the considerably reduced metallization area. As expected, a pressure of 20 MPa seems to form an acceptable bonding joint. Compared to the samples with full-area metallization, the fractures occurring in the RMS are almost always filled with solder, most likely because they cannot form cohesive bonds with the ceramic material in the unmetallized areas. It is noteworthy that, despite the high pressures, no fractures were found in the ceramic. Magnification: 20×.

**Figure 9:**
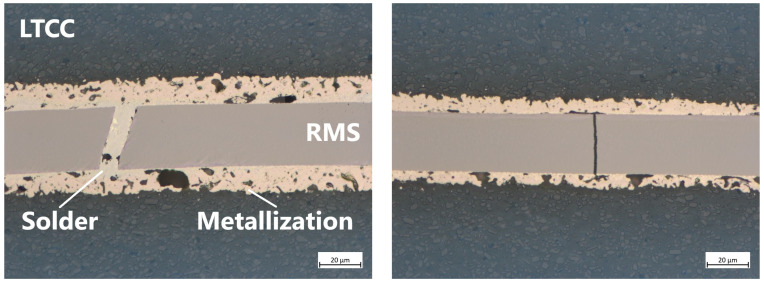

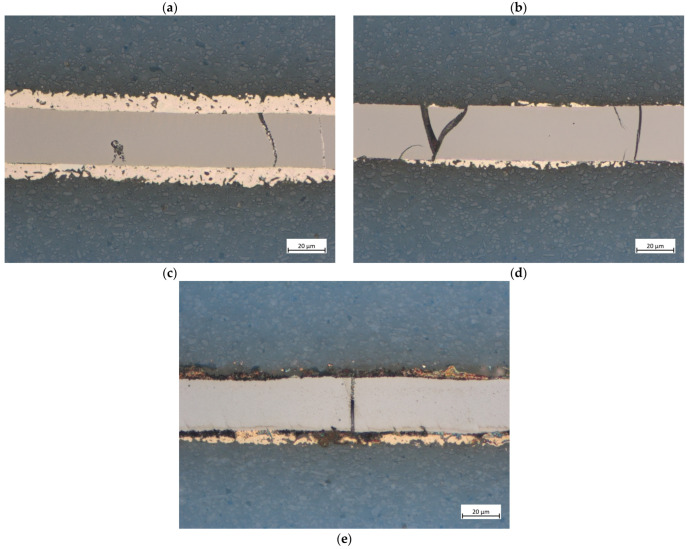
Detailed pictures of LTCC samples L6–L10 with Pd/Ag metallization on four corner pads of size 0.5 mm × 0.5 mm (blue: LTCC material; gray: RMS after reaction; white-yellow: solder; red-yellow: metallization). Compared to the previous samples, the applied pressures of (**a**) 20 MPa, (**b**) 100 MPa, (**c**) 200 MPa, (**d**) 300 MPa, and (**e**) 400 MPa are significantly higher due to the considerably reduced metallization area. Magnification: 50×.

Figure 8 shows the stitched images of samples L6–L10 with Pd/Ag metallization on four corner pads of size 0.5 mm × 0.5 mm, bonded under significantly higher pressures of approximately 20 MPa (L6), 100 MPa (L7), 200 MPa (L8), 300 MPa (L9), and 400 MPa (L10) due to the much smaller metallization area. Figure 9 presents more detailed images at 50× magnification, representative of the respective samples. Fundamentally, similar observations can be made here as with the samples with full-area metallization. According to previous results, a bonding pressure of 20 MPa already seems to lead to good results (Figure 8a and Figure 9a). Thus, there are few gaps in the pad area, and the separation between solder and metallization is still clearly visible. While cracks in the RMS in the pad area mostly remain unfilled, the solder is almost always drawn into the crack at the unmetallized areas.

In the medium pressure range of 100 MPa (Figure 8b and Figure 9b) and 200 MPa (Figure 8c and Figure 9c), the number of cracks increases significantly, especially in the pad area, while the thicknesses of the solder and metallization shrink. With a further increase in pressure to 300 MPa (Figure 8c and Figure 9c) or 400 MPa (Figure 8d and Figure 9d), these effects are further intensified. Additionally, in these cases, the metallization is also damaged, but neither separation of the RMS nor cracks in the LTCC could be observed.

### 3.2. EDX Analysis

**Figure 10:**
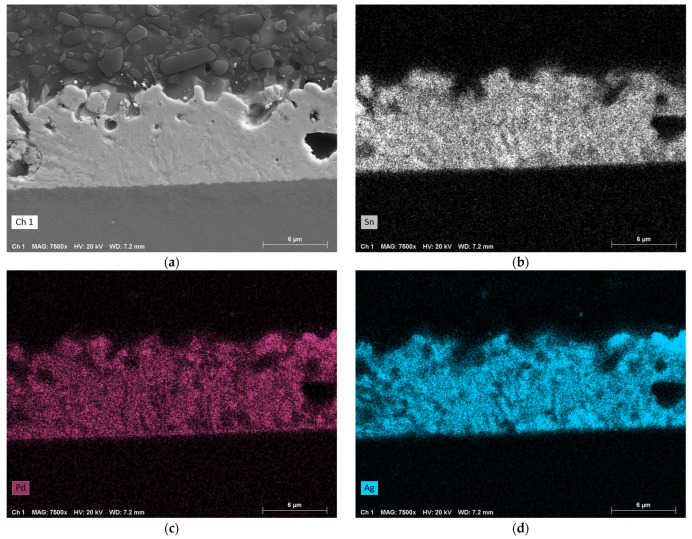

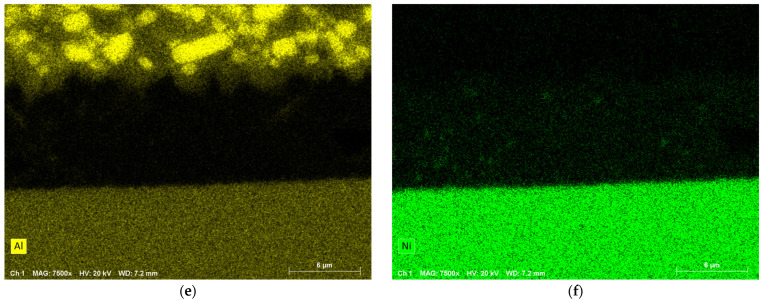
EDX analysis of a sample that was bonded under a pressure of approximately 2 MPa (bottom: RMS; top: LTCC; center: intermixing zone). (**a**) SEM image in SE mode. (**b**) EDX map of Sn. (**c**) EDX map of Pd. (**d**) EDX map of Ag. (**e**) EDX map of Al. (**f**) EDX map of Ni. Magnification: 7500×.

**Figure 11:**
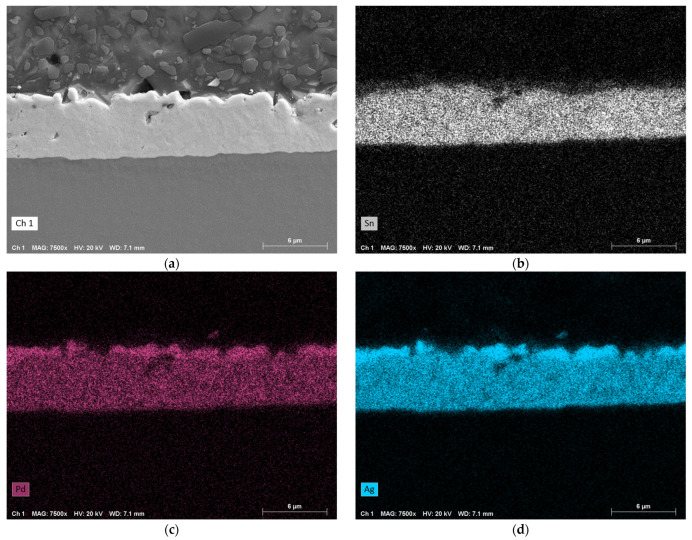

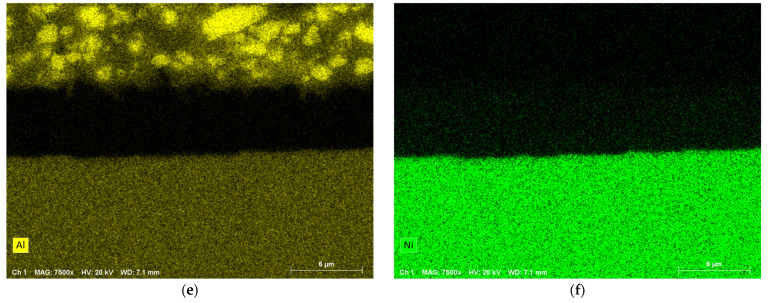
EDX analysis of a sample that was bonded under a pressure of approximately 11 MPa (bottom: RMS; top: LTCC; center: intermixing zone). (**a**) SEM image in SE mode. (**b**) EDX map of Sn. (**c**) EDX map of Pd. (**d**) EDX map of Ag. (**e**) EDX map of Al. (**f**) EDX map of Ni. Magnification: 7500×.

**Figure 12:**
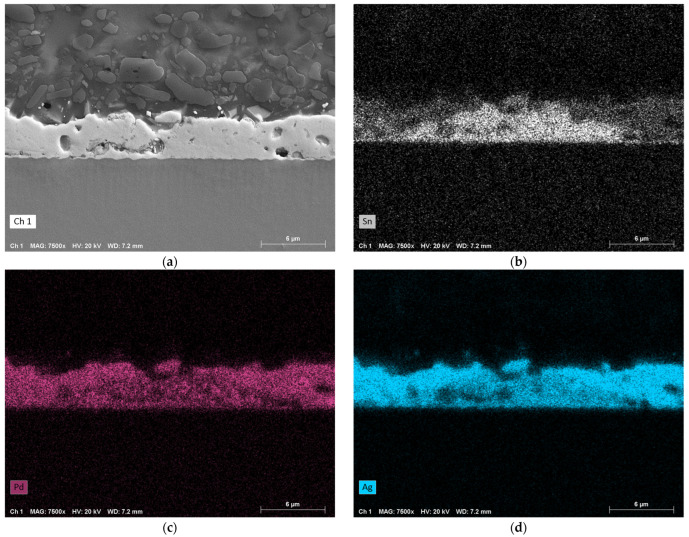

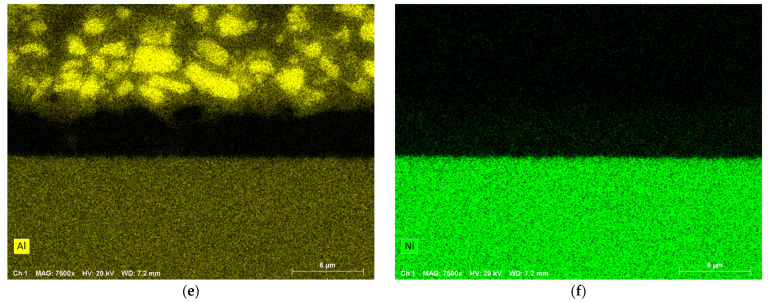
EDX analysis of a sample that was bonded under a pressure of approximately 22 MPa (bottom: RMS; top: LTCC; center: intermixing zone). (**a**) SEM image in SE mode. (**b**) EDX map of Sn. (**c**) EDX map of Pd. (**d**) EDX map of Ag. (**e**) EDX map of Al. (**f**) EDX map of Ni. Magnification: 7500×.

The distribution of some of the occurring elements in the upper interface between the RMS and the metallization was analyzed by EDX for bonding pressures of 2 MPa, 11 MPa, and 22 MPa. Figure 10, Figure 11 and Figure 12 provide detailed images of this zone, presented as SEM images in secondary electron contrast (SE) mode, along with EDX maps for the elements Sn (from RMS coating), Pd (from metallization), Ag (predominantly from metallization), Al (from RMS), and Ni (from RMS). Note that the LTCC material contains Al_2_O_3_ in comparatively high concentrations, making the very low concentration of Al in the Sn layer barely detectable.

Figure 10 illustrates the EDX analysis for a bonding pressure of 2 MPa. Due to the low pressure, the bonding zone is quite large and some unfilled gaps have formed. The three EDX maps show that Sn has not diffused into other areas after the exothermic reaction and remains confined to its original area. However, the metallization appears to have separated from the LTCC, as Pd and Ag are found to be almost uniformly distributed throughout the intermixing zone. Further, very small amounts of Al and Ni from the RMS have diffused into this layer. There is no difference in the concentration of these elements between the interfaces LTCC/solder or solder/RMS.

At a higher pressure of 11 MPa (Figure 11), the intermixing zone has shrunk to just over half its previous size. As previously observed, small amounts of Al and Ni from the RMS have diffused into the solder layer. The elements Sn, Pd, and Ag have again fully mixed together, but there is now a thin layer at the interface LTCC/solder with a lower concentration of Sn and higher concentration of Pd and Ag. No change can be observed at the interface solder/RMS.

With a further increase in pressure to 22 MPa (Figure 12), the intermediate layer shrinks further. While Al and Ni behave as in the previous case, the behavior of the other elements changes. Sn, Pd and Ag still intermix, but there are regions with high concentrations of Sn and low concentrations of Pd and Ag, and vice versa.

## 4. Discussion

The results of the configurations studied demonstrate that the bonding pressure during reactive bonding has a significant impact on the quality of the bonding zone. Furthermore, there are also differences between full-area and structured samples.

The light microscopic images reveal that insufficient bonding pressure leads to poor results. Numerous gaps form between the RMS and the metallization of the LTCC, with localized accumulations of solder. These gaps likely arise because the volume of the RMS shrinks by up to 12% after the transformation [24]. These gaps reduce the effective contact area between the RMS and the metallization, leading to poorer thermal conductivity [21]. It is assumed that this reduction also increases the electrical contact resistance and decreases the mechanical stability, both of which should be avoided to maintain a reliable connection. The application of pressure during the bonding process generally proves effective in reducing these gaps. However, in combination with the high temperatures exceeding 800 °C that occur during the exothermic reaction, significant thermo-mechanical stresses arise within the RMS due to the differing CTEs of Al (α_Al_ = 23 × 10^−6^ K^−1^) and Ni (α_Ni_ = 13 × 10^−6^ K^−1^), ultimately leading to the formation of cracks. These cracks mostly remain unfilled, as the solder tends to adhere to the metallization. Excessive pressures above 30 MPa in the case of full-area samples, on the other hand, are detrimental to the process and should be avoided. They are responsible for the destruction of the metallization or even for cracks in the ceramic material, probably caused by the initial expansion of the RMS during the exothermic reaction.

The comparison between full-area and samples with interconnects also reveals some differences. As in the case of the full-area samples, increasing pressure results in more cracks in the RMS for the structured samples. However, these cracks are more frequently filled with solder in the unmetallized areas, as Sn does not adhere to the ceramic and is drawn into the cracks by the combined effects of surface tension and capillary action, thereby additionally stabilizing the RMS. Furthermore, despite significantly higher pressures of up to 400 MPa, fewer damages were found. This is likely because, on the one hand, the pressure is concentrated on the small, metallized pads, while most of the RMS experience effectively no load due to the lack of contact (compare Figure 5). On the other hand, the RMS can expand into these tiny unmetallized gaps between it and the LTCC during the exothermic reaction, resulting in less stress.

The EDX analysis of the fully metallized samples shows that a certain pressure must be maintained for proper bonding. At low pressure of 2 MPa, there is still a gap between the RMS and the chip or substrate surface. Therefore, it seems there is an accumulation of solder at individual contact points, driven by surface tension. It is believed that the large amounts of solder at these points are able to completely dissolve the metallization. Another possible reason for this could is the significantly lower thermal conductivity of LTCC (3.3 W·m^−1^·K^−1^) compared to silicon (150 W·m^−1^·K^−1^) or copper (380 W·m^−1^·K^−1^), which leads to less heat being conducted away from the reaction zone. As a result, the individual layers are likely to approach the maximum reaction temperature of the RMS more closely than in other systems. At higher pressures of 11 MPa and 22 MPa, such dissolution of the metallization was not observed. Although a large portion dissolves into the solder layer, a strip with a comparatively high concentration remains intact, and there is full-area contact across the entire bonding zone (compare Figure 7b and Figure 7c). Finally, it can be observed that Al and Ni from the RMS diffuse evenly into the solder layer, although in low concentrations.

## 5. Conclusions

The use of an RMS as a localized heat source is already a well-established process in bonding silicon or metals. However, transferring this technology to ceramic LTCC substrates is complicated due to their specific properties, such as lower thermal conductivity and higher roughness. One aspect that greatly influences the bonding process is the applied bonding pressure. Therefore, LTCC samples with full-area and partial Pd/Ag metallization were bonded using various pressures between 2 MPa and 400 MPa, and cross-sections of the bonding zones were examined by light microscopy, SEM, and EDX. The analyses indicate an optimal pressure range for the bonding process, likely in the range between 10 MPa and 20 MPa.

For samples with full-area metallization, low bonding pressures result in poor wetting behavior of the solder, which accumulates in a few localized areas. Large-area gaps are observed, reducing the effective contact area between the chip and the substrate, thereby negatively affecting the mechanical stability and contact resistance. Conversely, excessive pressure improves the adhesion of the metallization to the RMS but causes it to detach from the LTCC base material and increases the number of cracks in the RMS. Additionally, pressures exceeding 30 MPa increase internal stress within the RMS, causing it to break into separate sections. Furthermore, such high pressures appear to enhance the dissolution of the metallization within the solder, likely reducing the chance of forming a proper IMC.

The EDX analysis supports the hypothesis of an optimal pressure range. At 2 MPa, the elements Pd and Ag from the metallization dissolve nearly completely into the solder due to the inhomogeneous distribution of the solder. At a slightly higher pressure of 11 MPa, this intermixing also occurs, but the metallization itself remains intact and there is a full-area contact across the entire bonding zone. Further, the intermixing layer between the RMS and the LTCC is more densely packed. At 22 MPa, similar observations are made, with areas showing accumulations of Sn and lower concentrations of Pd and Ag, and vice versa.

For the samples with interconnects, high pressures do not seem to have as severe an impact. Although more cracks are formed in the RMS with increasing pressure, neither complete fragmentation of the RMS nor cracks in the LTCC occur even at extremely high pressures of up to 400 MPa. Due to the partial metallization, there are small gaps between the LTCC and the RMS, into which the RMS can likely expand during the exothermic reaction before subsequently shrinking to its final form.

However, not only the bonding pressure but also the temperature during the exothermic reaction has a significant influence on the intermixing of the particular layers and the formation of cracks within the RMS. Additionally, a thicker solder layer may also help to fill any resulting cracks, though the range of different types of commercially available reactive foils is very limited. Alternatively, direct deposition of such reactive layers onto the substrate or chip is possible, allowing for the variation in individual parameters, such as stack or nanolayer thickness, or solder thickness. Future research could therefore investigate the combined influence of these parameters and the bonding pressure to further optimize the bonding process.

## Figures and Tables

**Table 1:** Overview of the sample types that were used for the experiments.

Configuration	Base Material	Substrate Size [mm^2^]	Chip Size [mm^2^]	Type of Metallization	Effective Bonding Area [mm^2^]
L1–L5	LTCC	15 × 3	3 × 3	Pd/Ag	3 × 3
L6–L10	LTCC	15 × 3	3 × 3	Pd/Ag	4 × 0.5 × 0.5

**Table 2:** Bonding profiles and resulting bonding pressures for the LTCC samples. The preheat and peak temperatures were set identically on both the chuck and the tool. Due to the much smaller bonding area in the case of samples with four corner pads, the pressure is many times higher.

Configuration	Preheat Temperature [°C]	Peak Temperature [°C]	Force [N]	Metallization Area [mm^2^]	Bonding Pressure [MPa]
L1	80	200	20	3 × 3	2
L2	80	200	100	3 × 3	11
L3	80	200	200	3 × 3	22
L4	80	200	300	3 × 3	33
L5	80	200	400	3 × 3	44
L6	80	200	20	4 × 0.5 × 0.5	20
L7	80	200	100	4 × 0.5 × 0.5	100
L8	80	200	200	4 × 0.5 × 0.5	200
L9	80	200	300	4 × 0.5 × 0.5	300
L10	80	200	400	4 × 0.5 × 0.5	400

## Data Availability

The original contributions presented in this study are included in the article. Further inquiries can be directed to the corresponding author.
